# Walking the interactome for candidate prioritization in exome sequencing studies of Mendelian diseases

**DOI:** 10.1093/bioinformatics/btu508

**Published:** 2014-07-30

**Authors:** Damian Smedley, Sebastian Köhler, Johanna Christina Czeschik, Joanna Amberger, Carol Bocchini, Ada Hamosh, Julian Veldboer, Tomasz Zemojtel, Peter N. Robinson

**Affiliations:** ^1^Mouse Informatics Group, The Wellcome Trust Sanger Institute, Wellcome Trust Genome Campus, Hinxton, Cambridgeshire CB10 1SA, UK, ^2^Institute for Medical Genetics and Human Genetics, Charité-Universitätsmedizin Berlin, Augustenburger Platz 1, 13353 Berlin, ^3^Genome Informatics Department, Institute of Human Genetics, University Hospital Essen, University of Duisburg-Essen, Hufelandstr. 55, 45122 Essen, Germany, ^4^McKusick-Nathans Institute of Genetic Medicine, John Hopkins University School of Medicine, Baltimore, MD 21205, USA, ^5^Department of Mathematics and Computer Science, Institute for Bioinformatics, Freie Universität Berlin, Takustrasse 9, 14195 Berlin, Germany, ^6^Institute of Bioorganic Chemistry, Polish Academy of Sciences, 61-701 Poznan, Poland, ^7^Berlin-Brandenburg Center for Regenerative Therapies, Charité-Universitätsmedizin Berlin, Augustenburger Platz 1, 13353 Berlin and ^8^Max Planck Institute for Molecular Genetics, Ihnestrasse 73, 14195 Berlin, Germany

## Abstract

**Motivation**: Whole-exome sequencing (WES) has opened up previously unheard of possibilities for identifying novel disease genes in Mendelian disorders, only about half of which have been elucidated to date. However, interpretation of WES data remains challenging.

**Results**: Here, we analyze protein–protein association (PPA) networks to identify candidate genes in the vicinity of genes previously implicated in a disease. The analysis, using a random-walk with restart (RWR) method, is adapted to the setting of WES by developing a composite variant-gene relevance score based on the rarity, location and predicted pathogenicity of variants and the RWR evaluation of genes harboring the variants. Benchmarking using known disease variants from 88 disease-gene families reveals that the correct gene is ranked among the top 10 candidates in ≥50% of cases, a figure which we confirmed using a prospective study of disease genes identified in 2012 and PPA data produced before that date. We implement our method in a freely available Web server, ExomeWalker, that displays a ranked list of candidates together with information on PPAs, frequency and predicted pathogenicity of the variants to allow quick and effective searches for candidates that are likely to reward closer investigation.

**Availability and implementation**: http://compbio.charite.de/ExomeWalker

**Contact**: peter.robinson@charite.de

## 1 INTRODUCTION

The identification of causative disease genes in Mendelian disorders has contributed greatly to our understanding of gene functions and biological pathways in rare and common disease (Antonarakis and Beckmann, 2006). With the development of whole-exome sequencing (WES), the pace of identification of novel disease genes has accelerated (Gilissen *et al.*, 2011) to the extent that groups such as the International Rare Disease Research Consortium has set out the goal of comprehensive discovery of the molecular etiologies of all rare diseases to enable molecular diagnosis for all affected individuals by the year 2020 (Baxter and Terry, 2011).

Before WES, most gene discovery projects made use of linkage analysis or association studies, which typically identified genomic intervals of 0.5–10 cm containing up to 300 genes (Botstein and Risch, 2003; Glazier *et al.*, 2002). Numerous computational procedures have been developed to prioritize candidate genes in the intervals and guide DNA sequencing efforts (reviewed in Moreau and Tranchevent, 2012). Although WES provides sequence information for the great majority of targeted exon sequences, the need for prioritization remains. An individual exome typically contains >30 000 variants as compared with the genomic reference sequence, thousands of which are predicted to lead to non-synonymous amino acid substitutions, alterations of conserved splice site residues or small insertions or deletions. Typical analysis strategies have relied on the characteristics of the variants, focusing on rare variants that are predicted to be pathogenic (Robinson *et al.*, 2011), but even after such filtering, around ∼100–1000 candidate disease-causing variants are found in a single WES dataset, and additional methods are needed to predict which of them may have serious functional consequences and prioritize them for validation (Li *et al.*, 2013; Pelak *et al.*, 2010). Because each genome harbors ∼100 genuine loss-of-function (LOF) variants with ∼20 genes completely inactivated (MacArthur *et al.*, 2012), a purely variant-based prioritization of candidate genes in WES studies will be limited in its ability to correctly identify the true disease gene.

Previous gene prioritization strategies for prioritizing genes in linkage studies evaluated one or more characteristics of the genes, including functional annotation, gene-expression data or sequence-based features (Tranchevent *et al.*, 2011). Strategies to prioritize candidate genes in exome sequencing studies can also exploit the variant data itself in an attempt to improve prioritization of Mendelian disease genes, somatic mutations in cancer and others. A number of tools and pipelines have been developed that exploit sophisticated variant filtering strategies. The tools combine filtering steps that exclude common variants and retain only variants that are predicted likely pathogenic using tools such as MutationTaster (Schwarz *et al.*, 2010), and then exploit sequences from multiple unrelated individuals with the sought-after disease to search for genes mutated in most or all of the individuals, as well as linkage or pedigree analysis (Coutant *et al.*, 2012; Li *et al.*, 2012; Santoni *et al.*, 2014; Sifrim *et al.*, 2012; Yandell *et al.*, 2011; Zhang *et al.*, 2013). Recently, approaches have been introduced that combine variant impact prediction with gene prioritization. The eXtasy algorithm uses genomic data fusion to integrate variant impact prediction, haploinsufficiency prediction and phenotype-specific gene prioritization (Sifrim *et al.*, 2013). The Exomiser implements PHIVE, PHenotypic Interpretation of Variants in Exomes, an algorithm that integrates the calculation of phenotype similarity between human diseases and genetically modified mouse models, with evaluation of the variants according to allele frequency, pathogenicity and mode of inheritance (Robinson *et al.*, 2014). FunSeq intersects regions of the genome that are likely to be sensitive to mutations with an analysis for variants that disrupt transcription-factor binding sites (Khurana *et al.*, 2013). Each of these algorithms essentially seeks genes or genomic regions that are both relevant to the disease under investigation and also harbor variants likely to be pathogenic. We therefore reasoned that a key factor in exome prioritization algorithms is to intersect the results of variant analysis with a method that can prioritize genes according to their potential relevance.

The analysis of protein interaction networks has been widely used for computational analysis of human disease (Barabási, 2007; Gonzalez and Kann, 2012). Typically, proteins do not act in isolation, but rather perform their functions cooperatively within a network of functionally related proteins. That is, groups of functionally related proteins may physically interact with one another and thereby form a ‘molecular nanomachine’ that mediates a particular biological function at cellular or systems level. A protein–protein interaction (PPI) may be defined as a specific physical contact with molecular docking between proteins that occurs in cells or in a living organism *in vivo* (De Las Rivas and Fontanillo, 2010). Currently, data on >100 000 PPIs in humans are available (Schaefer *et al.*, 2013), derived from experimental methods including the yeast two-hybrid system and tandem affinity purification. In this work, we make use of data from the search tool for the retrieval of interacting genes/proteins (STRING) (Franceschini *et al.*, 2013), which contains not only PPIs but also indirect (functional) associations derived from genomic context, high-throughput experiments, conserved coexpression and text-mining. We will refer to this network as the protein–protein association (PPA) network. The complete set of all such interactions and associations has been referred to as the interactome, and with the increased quantity and quality of such data, analysis of the protein interactome offers an important resource for systems-level understanding of cellular processes.

The interactome has also become an important resource for the computational prioritization of disease genes (Moreau and Tranchevent, 2012). The main assumption of these methods is that genes linked to diseases with similar or even identical phenotypic manifestations will in many cases code for genes that interact in specific subnetworks within the larger interactome. Therefore, lists of candidate genes can be prioritized according to the vicinity of the candidates genes within the interactome to other known members of a given disease-gene family. Initial efforts to rank disease genes exploited the presence of direct interactions (Oti *et al.*, 2006) or the length of the shortest path of interactions leading from a candidate gene to a known disease gene (George *et al.*, 2006). We have shown that a global network measure of distance in the protein–protein interaction network obtained by random walk analysis, substantially improves candidate–gene prioritization, including the search for direct neighbors of other disease genes (Köhler *et al.*, 2008). In fact, it was shown that random-walk approaches outperform other gene-prioritization methods (Navlakha and Kingsford, 2010). In this work, we test the hypothesis that random-walk analysis of the protein interactome can improve prioritization of candidate disease genes in exome sequencing studies.

## 2 METHODS

### 2.1 Protein–protein and functional interaction data

The PPA network is represented by an undirected graph with nodes representing the genes and edges representing the mapped interactions of the proteins encoded by the genes. Data were taken from STRING (Search Tool for the Retrieval of Interacting Genes/Proteins) version 9.05, which contains experimental, predicted and transferred protein–protein interactions, together with interactions obtained through text mining (Franceschini *et al.*, 2013). Only high-confidence interactions (score at least 0.7) were used.

### 2.2 Disease-gene families

A disease-gene family is defined here as a group of genes in which a mutation in any one of the genes leads to a clinically similar disorder. Thus, a disease-gene family comprises the genes associated with some genetically heterogeneous disease. In this work, we have used data on the phenotypic series from Online Mendelian Inheritance in Man (OMIM) (Amberger *et al.*, 2011) of March 2013. Each phenotypic series provides a view of genetic heterogeneity of similar phenotypes across the genome.

### 2.3 Simulation of whole-exome and disease data

To validate our methodology, we developed a simulation strategy based on adding known disease-causing mutations from the Human Gene Mutation Database (HGMD) into either one of 1092 unaffected whole-exome files in variant call format (VCF) from the 1000 Genomes Project (1000 Genomes Project Consortium *et al.*, 2012) or 144 in-house exomes. The 1000 Genomes Project individual whole-exome files were extracted from the integrated call sets (October 12, 2012 release) using tabix (Li, 2011) version 0.2.6 and vcftools (Danecek *et al.*, 2011) version 0.1.9. From the initial 233 phenotypic series involving 1356 genes, we eventually tested 88 series that contained at least four genes and with known HGMD mutation(s) for the disease described in the phenotypic series, corresponding to 285 genes. For autosomal dominant (AD) diseases, one heterozygous mutation was added, and for autosomal recessive (AR) diseases, one homozygous mutation was added to the whole-exome files.

### 2.4 Whole-exome analysis and filtering

For each of the simulated exomes, we used an exome analysis pipeline to filter variants according to rarity, predicted pathogenicity and conformance with the expected mode of inheritance. To filter variants according to rarity, information concerning population minor allele frequency (MAF) of variants was derived from the database of single nucleotide polymorphisms (dbSNP) (NCBI Resource Coordinators, 2013) and from the Exome Variant Server (2013). For this work, the maximum population frequency of a variant was taken to be its maximal reported frequency in any data source. For the dbSNP data, only the reported frequencies from Phase I 1000 Genome Project variants were included. In addition, ExomeWalker scores variants according to the MAF as previously described (Robinson *et al.*, 2014) to give a frequency score between 1 and 0 for variants with a MAF between 0 and 2%, with more common variants receiving a score of 0. In all simulations reported in this work, variants with a MAF >1% were excluded.

In a typical whole-exome analysis, many of the variants have no available frequency data in public databases for assessment. Hence, for the simulations involving 1000 Genomes Project-based exomes, we did not make use of the 1000 Genomes Project frequency data, as this would lead to an unfair advantage because each of the non–disease-associated variants would have frequency data available for filtering and prioritization.

Variants in the VCF files (which are defined using chromosomal coordinates) were then annotated at transcript level using Jannovar (Jäger *et al.*, 2014). To filter variants according to predicted pathogenicity, a variant score was calculated for each variant. First of all, off-target variants (those not located in protein coding sequences or in splice sites) were given a score of zero and removed. Secondly, non-synonymous variants leading to the substitution of an amino acid residue were scored according to the most deleterious prediction of SIFT (Ng and Henikoff, 2002), Polyphen2 (Adzhubei *et al.*, 2010) or MutationTaster (Schwarz *et al.*, 2010). These predictions were extracted from dbNSFP (Liu *et al.*, 2011). Links between genes and Mendelian diseases were extracted from data of the Online Mendelian Inheritance in Man resource (Amberger *et al.*, 2011). In some cases, no predictions were available from any of these three sources, and a pathogenicity prediction of 0.6 was assigned. This value was arrived at during optimization for another exome prioritization tool (Robinson *et al.*, 2014) and represents a compromise between assuming novel variants are non-pathogenic or fully pathogenic. For other classes of variants, pathogenicity scores were assigned as previously described (Robinson *et al.*, 2014). Future versions of ExomeWalker will look to incorporate a single measure of pathogenicity for all types of variants such as CADD scores (Kircher *et al.*, 2014).

For variants that pass the filtering steps, a variant score is assigned for prioritization, which is simply the product of this pathogenicity score and the frequency score described above.

### 2.5 Random walk analysis

The random walk on graphs (Can *et al.*, 2005) is defined as an iterative walker’s transition from its current node to a randomly selected neighbor starting at a given source node, *s*. Here, we used a variant of the random walk in which we additionally allow the restart of the walk in every time step at node s with probability *r*. Formally, the random walk with restart (RWR) is defined as

(1) $$p_{t+1}=(1-r)Wp_{t}+rp_{0}$$

The transition matrix **W** is the column-normalized adjacency matrix of the graph, and $p_{t}$ is a vector in which the *i*^th^ element holds the probability of being at node *i* at time step *t*.

In our application, the initial probability vector $p_{0}$ was constructed such that equal probabilities were assigned to the nodes representing members of the disease, with the sum of the probabilities equal to 1. This is equivalent to letting the random walker begin from each of the known disease genes with equal probability. Candidate genes were ranked according to the values in the steady-state probability vector $p_{\infty }$. While it is possible to obtain $p_{\infty }$ by explicitly calculating Equation (1) until convergence, we instead solve the equation $p_{\infty }=(1-r)Wp_{\infty }+rp_{0}$ to obtain

(2) $$p_{\infty }=r{\left(I-(1-r)W\right)}^{-1}p_{0}$$

By precalculating the matrix $r{\left(I-(1-r)W\right)}^{-1}$, we can perform random walk analysis as a simple matrix multiplication of the vector $p_{0}$ in $O(n^{2})$ time, where *n* is the number of genes in the network. Therefore, denoting $r{\left(I-(1-r)W\right)}^{-1}$ by **R**, we can calculate the result of the random walk analysis by a simple matrix multiplication $p_{\infty }=Rp_{0}$. We can further simplify the calculations by noting that most of the elements of the vector $p_{0}$ are zero, with only the elements representing the *m* seed genes having the non-zero value $\frac{1}{m}$. Denoting the set of the indices of these elements as {j′}, then it is easy to see that only the corresponding columns of **R** contribute to the final values of $p_{\infty }$, whose *i*^th^ element can be given as

(3) $$p_{\infty }[i]=\frac{1}{m}\sum_{j\in \left\{j\prime \right\}}R[\;j,i]$$

That is, to get element *i* in $p_{\infty }$, we need only to take the sum of the products of the non-zero elements of $p_{0}$ with the corresponding elements of column *i* of **R**. The computational complexity of the random walk analysis in Equation (1) is dominated by the matrix-vector multiplications in each step, which is $O(n^{2})$ for an *n* × *n* matrix. In contrast, our method requires precomputation of one matrix inversion, but the actual calculation of $p_{\infty }$ is O(mn) with m≪n, as there are O(m) operations to calculate Equation (3), which has to be done for each of the *n* elements of $p_{\infty }$.

$p_{\infty }$ is a probability vector, and all its entries sum to unity. For the purposes of exome analysis, only those genes that have rare predicted pathogenic variants are considered. For the analysis described in this article, we chose a value of 0.7 for the restart probability *r*.

### 2.6 ExomeWalker score

Finally, a gene is assigned a combined ExomeWalker score, which is a combination of the random walk score and the best scoring variant in that gene. In the case of AR inheritance under a compound heterozygous model, the variant score is taken to be the average of the two highest scoring variants. Logistic regression on a training set of 20 000 disease variants and 20 000 benign variants was run through the Waikato Environment for Knowledge Analysis (WEKA) (Hall *et al.*, 2009) data-mining package to generate the optimal way of combining the variant and random walker scores into a final ExomeWalker score. A 10-fold cross validation was used to train and test the model, and the average of the 10 models was used for the final algorithm. Receiver operating characteristic analysis on the test datasets gave an average area under the curve of 0.96 for the ExomeWalker score compared with 0.78 for the variant score and 0.9 for the random walk score. This final ExomeWalker score gives a measure from 0 to 1 of how close the gene is to known disease-associated genes in the interactome and how rare and pathogenic are the variants in the gene.

### 2.7 Benchmarking of ExomeWalker

For the simulated exomes involving known disease variants in 285 genes from 88 phenotypic series, we performed 5000 analyses per experiment. ExomeWalker was run using the other genes in the phenotypic series as seed genes for the random walk. Genes were ranked by either the variant score or the ExomeWalker score. We then compared performance by assessing how often the known disease gene was recovered as the top hit or in the top 10 or 50 candidates. An ordinal ranking method was used where equal scoring genes are resolved arbitrarily but consistently by assigning a unique rank to each of the ties. In our case, we simply sort the equally scored genes alphabetically and assign the ranks. This corresponds to the real life use case where a researcher would have to take each of the equally scored top candidates and investigate each one by one for causality by further experimentation or for further candidacy by reviewing the literature/databases using their expert knowledge.

## 3 RESULTS

In this work, we have implemented an algorithm for prioritizing candidate genes in WES studies by searching for rare variants with predicted pathogenicity in genes located in the vicinity of phenotypically related genes in a functional interaction network. We constructed a PPA network based on 210 945 associations among 12 511 human genes using high-confidence interactions in the STRING database (Franceschini *et al.*, 2013). We implemented a global distance measure based on RWR to define similarity between genes within this interaction network (Köhler *et al.*, 2008). The RWR algorithm ranks genes on the basis of their similarity to known or suspected disease genes. In our benchmarking, we use phenotypic series from the OMIM resource (Amberger *et al.*, 2011) to define disease-gene families. Users of ExomeWalker can either use these same disease-gene families or enter their own list of genes that are known or suspected to be associated with the disease being studied.

In parallel, variants from an exome are annotated and the frequency and predicted pathogenicity are evaluated. Candidate genes with rare, potentially pathogenic variants are then prioritized using both the results of variant evaluation and the vicinity in the PPA network of the genes harboring the variants to the seed genes (see Section 2 for details).

**Fig. 1.**
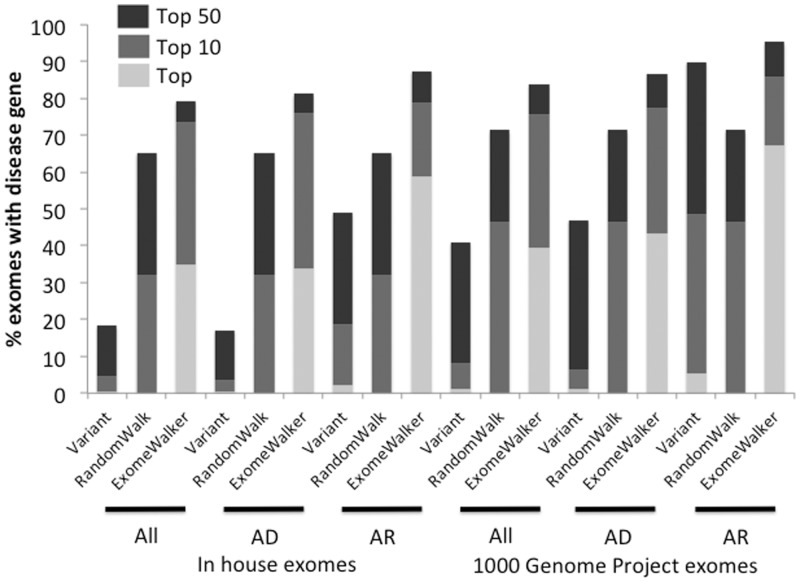
Performance of ExomeWalker using STRING v9.05 as the source of interactome data. The bars show the percentage of exomes where the true disease gene is identified as the top hit or in the top 10 or 50 results. Either in-house or 1000 Genomes Project exomes were used. All exomes are filtered to remove synonymous, intergenic and intronic variants except for those in splice sites. In addition, variants with a MAF > 1% are excluded. Results are shown without (All) or with an AD or AR inheritance model applied. Ranking is either by Variant scoring that combines MAF and predicted pathogenicity, RWR analysis alone or ExomeWalker scoring that additionally includes evidence of protein–protein associations with other genes linked to the disease

Figure 1 shows the results of our analysis using STRING v9.05 as the source of interactome data. Analysis was performed by adding known disease variants to either in-house exomes or 1000 Genome Project exomes and with and without the appropriate inheritance model for the disease being tested.

**Fig. 2.**
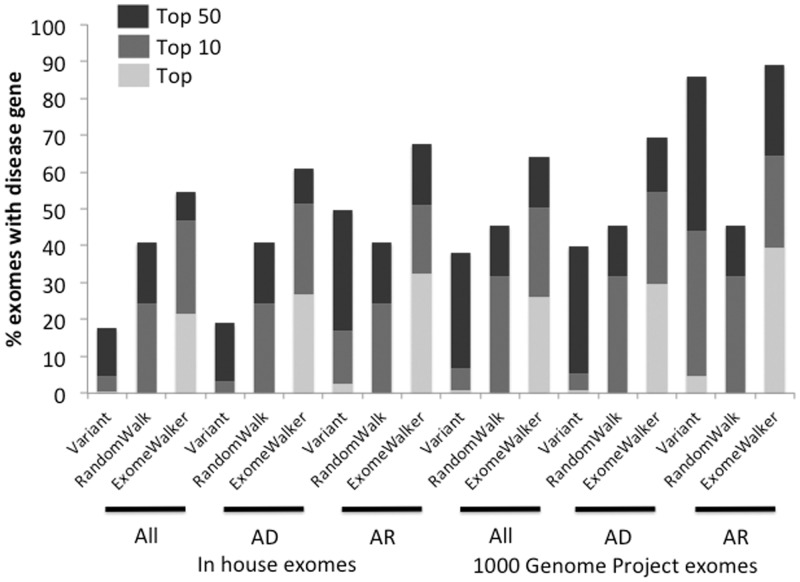
Performance of ExomeWalker using STRING v9.05 without text-mined associations as the source of interactome data. Abbreviations are as in Figure 1

The results show a substantial performance increase when including the random walk measure of protein–protein interactions with other genes associated with the disease as compared with either the variant score or the RWR score alone. For example, 39.4% of the tested exomes contained the known disease gene as the top hit for the 1000 Genomes Project–based simulations compared with 1.4% when just using variant pathogenicity and frequency to assess candidacy. This is out of an average of 907 postfiltered genes and 97.1% of the disease genes are kept during this filtering step. This increases to 43.5 and 67.3% for the AD and recessive models out of an average of 632 and 374 postfiltered genes, respectively. Similar performance and gains are seen when adding the disease variants to our in-house exomes that contain many more postfiltered genes (1141 on average for no inheritance model). The correct gene was present within the top 10 ranked candidates in nearly 75% of the simulations using in-house exomes. As shown in Figures 1 and 2, a large proportion of the performance comes from the random walk prioritization of the filtered exome candidates with the addition of variant pathogenicity and frequency data adding a further 5–10% increase.

STRING includes text-mined associations between genes and it is possible some of these associations may come from publications describing two genes being associated with the same disease, rather than the biological associations we are trying to detect with our simulation studies. To allow for this, we repeated the analysis with a version of STRING where all text-mined associations had been removed (Fig. 2).

As expected, there is a drop in performance compared with including the text-mined association but ExomeWalker still shows a substantial improvement over purely variant-based measures of candidacy. For example, for the 1000 Genomes Project exomes with no inheritance model, the ExomeWalker performance drops from 39.4 to 26.1%, having the correct gene as the top hit but this is still 24.7% higher than with the variant-based scoring alone.

This strategy of removing all text-mined associations will have removed many genuine interactions that would be useful for prioritization of a novel disease–gene association. We expect the real performance of ExomeWalker in such cases to lie somewhere in-between that seen in Figures 1 and 2. To gain a realistic estimate of the performance of our method on new data, we identified 19 disease–gene associations that had been identified in 2012 that belong to one of the phenotypic series and had a known variant in HGMD. We tested the performance of our method using a PPA dataset with data before the discovery of any of these genes (STRING v9.0). The results are summarized in Table 1. The true disease-causing gene was present within the top 10 prioritized genes in 10 of 19 cases (∼53%), similar to our results using large-scale simulations.

**Table 1.** List of 19 genes discovered during the year 2012 and for which a disease-causing mutation was listed in HGMD

Gene	ID	Disease gene family	Publication date	Variant	RandomWalk	ExomeWalker	Exomiser	eXtasy
*CHMP1A*	5119	Pontocerebellar hypoplasia	November 2012 (Mochida *et al.*, 2012)	3	66	3	1	^a^
*NMNAT1*	64802	Leber congenital amaurosis	September 2012 (Falk *et al.*, 2012)	61	14	61	52	99
*CEP135*	9662	Microcephaly, primary AR	May 2012 (Hussain *et al.*, 2012)	18	3	2	16	^b^
*KLHL3*	26249	Pseudohypoaldosteronism, type II	January 2012 (Boyden *et al.*, 2012)	4	131	5	2	1
*THRA*	7067	Hypothyroidism, congenital, non-goitrous	January 2012 (Bochukova *et al.*, 2012)	51	2	1	148	5
*TMEM5*	10329	Muscular dystrophy-dystroglycanopathy, type A	December 2012 (Vuillaumier-Barrot *et al.*, 2012)	19	298	19	19	^b^
*DDOST*	1650	Congenital disorders of glycosylation, type I	February 2012 (Jones *et al.*, 2012)	22	9	23	17	^b^
*PNPT1*	87178	Combined oxidative phosphorylation deficiency	November 2012 (Vedrenne *et al.*, 2012)	9	4	2	119	3
*DPM2*	8818	Congenital disorders of glycosylation, type I	October 2012 (Barone *et al.*, 2012)	3	1	1	2	4
*PACS1*	55690	Mental retardation, AD	December 2012 (Schuurs-Hoeijmakers *et al.*, 2012)	29	283	39	15^c^	^c^
*ADAR*	103	Aicardi-Goutieres syndrome	November 2012 (Rice *et al.*, 2012)	17	26	17	119^c^	^c^
*CABP2*	51475	Deafness, AR	October 2012 (Schrauwen *et al.*, 2012)	59	109	61	40	^a^
*DST*	667	Hereditary sensory and autonomic neuropathy	April 2012 Edvardson *et al.*, 2012	18	108	18	132	^b^
*VPS37A*	137492	Spastic paraplegia	July 2012 (Zivony-Elboum *et al.*, 2012)	11	318	11	9	10
*HOXC13*	3229	Ectodermal dysplasia	November 2012 (Lin *et al.*, 2012)	19	5	17	131	48
*KANSL1*	284058	Mental retardation, AD	April 2012 (Koolen *et al.*, 2012; Zollino *et al.*, 2012)	88	63	45	222	178
*GUCY2C*	2984	Diarrhea, congenital	April 2012 (Fiskerstrand *et al.*, 2012; Wu *et al.*, 2012)	18	4	1	124	13
*PFN1*	5216	Amyotrophic lateral sclerosis	August 2012 (Wu *et al.*, 2012)	46	68	50	166	91
*CHD8*	57680	Autism	December 2012 (O’Roak *et al.*, 2012a and b)	80	95	80	192	^b^

*Notes.* The first column shows the gene symbol and the second column shows the NCBI Entrez Gene ID. The third column provides the OMIM phenotypic series to which the gene was assigned after its identification as being causative for the disease. The next three columns show the rank obtained in ExomeWalker analysis using STRING v9.0, 1000 Genomes Project exomes and mode of inheritance filtering sorted by the variant, random walk or combined ExomeWalker score. Finally, we show the ranks obtained from the Exomiser and eXtasy tools that take an alternative approach of prioritizing by phenotype. The columns Variant, RandomWalk, ExomeWalker, Exomiser and eXtasy show the ranks of the gene by each method. The eXtasy rank was obtained after preprocessing.

^a^Variant lost during eXtasy prioritization.

^b^Deletion so not suitable for eXtasy prioritization.

^c^No phenotype annotations.

## 4 DISCUSSION

Computational candidate gene prioritization has matured into a field that has developed and benchmarked scores of algorithms that exploit and integrate complex and heterogeneous datasets including gene expression, sequence annotations, data mining, genetic sequences, functional annotations and protein–protein interaction networks (Aerts *et al.*, 2006; Lage *et al.*, 2007; Perez-Iratxeta *et al.*, 2002; Tranchevent *et al.*, 2011; Turner *et al.*, 2003). The fundamental algorithms have been improved and extended in many ways, such as including tissue-specificity in the analysis of the protein interactome (Magger *et al.*, 2012). Initial computational analysis of exome sequence data concentrated on filtering variants according to their population frequency, predicted pathogenicity and the presence of rare predicted-pathogenic mutations in multiple unrelated individuals with a certain rare disease (‘intersection’ strategy; Boycott *et al.*, 2013; Robinson *et al.*, 2011). However, it has become apparent that it remains difficult to identify novel disease genes or even known disease genes with WES because of the sheer number of candidate mutations; each genome is thought to harbor ∼100 genuine LOF variants with ∼20 genes completely inactivated (MacArthur *et al.*, 2012). Therefore, filtering on variant characteristics alone is not effective in situations where a single affected individual or only a small number of individuals are being investigated. Therefore, just as positional cloning approaches were limited by the availability of large well-characterized families, disease-identification studies by WES are often limited by the number of individual exome sequences available for variant intersection. For this reason, candidate gene prioritization methods have recently begun to be applied to exome analysis. With positional cloning, prioritization would be applied to all genes located within the linkage interval; with exome studies, prioritization is applied to all genes that harbor rare, potentially pathogenic mutations. In both settings, the number of genes may be in the hundreds. Recently, exome prioritization methods have been introduced that exploit data fusion, phenotypic data and model organism phenotype data (Robinson *et al.*, 2014; Sifrim *et al.*, 2013). Random-walk analysis of protein–protein interaction data has been shown to be a powerful approach to gene prioritization in the setting of positional cloning projects (Köhler *et al.*, 2008; Navlakha and Kingsford, 2010). In this work, therefore, we have adapted our previous approach and tested its utility for exome analysis.

**Fig. 3.**
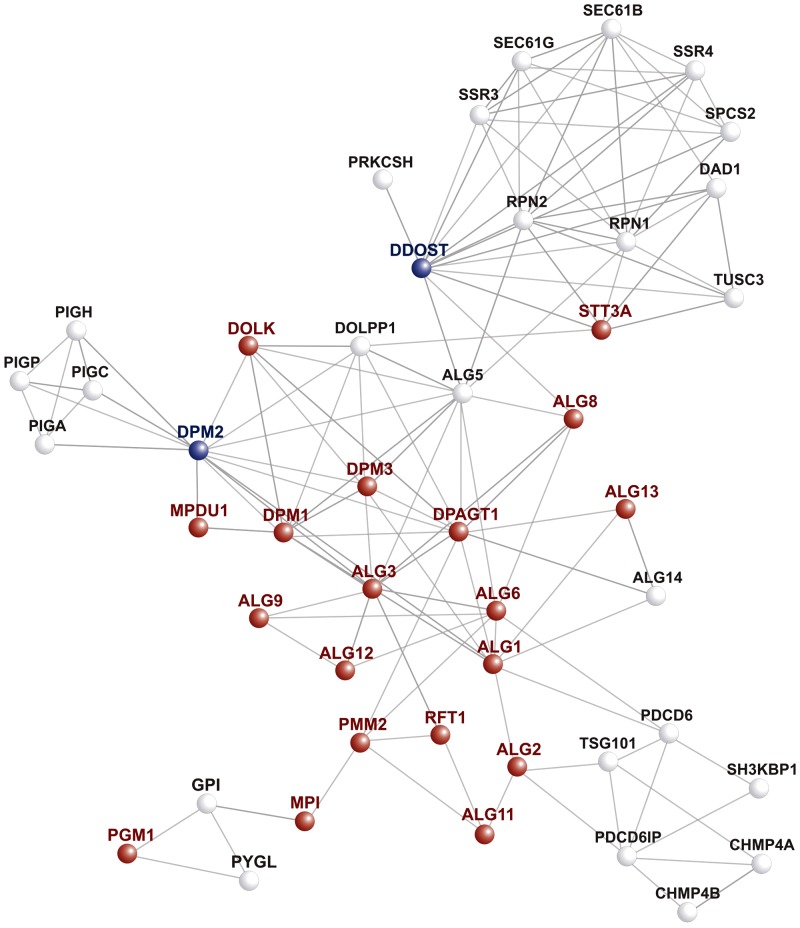
PPA network derived from congenital disorders of glycosylation, type I (CDG-I) seed genes. The candidate genes *DDOST* and *DMP2* (shown in blue) interact with multiple other CDG-I genes (shown as red nodes in the network) via paths of length one and two. The random walk methodology essentially integrates over all interaction paths between seed genes and a candidate gene to generate a similarity score. Although short paths such as those shown in the figure have the most influence on the score, other aspects of the global network structure are also taken into account (Köhler *et al.*, 2008)

Figure 3 illustrates the gene prioritization procedure in the case of *DDOST* and *DPM2*, components of the oligosaccharyltransferase complex that transfers a glycan chain to nascent proteins. Congenital disorders of glycosylation (CDG) are inherited AR diseases that impair *N*-glycosylation, and previously identified CDG disease genes were used to prioritize candidate genes including *DDOST* and *DPM2* in the simulations summarized in Table 1. It can be seen that *DDOST* has only two direct interactions with CDG seed genes and is at some distance from the others, leading to it only being ranked 23rd. However, *DPM2* has multiple direct and second-degree interactions with CDG genes leading to it being ranked as the top-ranked candidate in simulations.

In contrast, other genes did not achieve a high rank. For instance, *TMEM5*, mutations which were shown to be a cause of type A muscular dystrophy-dystroglycanopathy (Vuillaumier-Barrot *et al.*, 2012), was placed at rank 19 by our method. This gene has only one high-confidence association in the STRING database, with *PLAU* (plasminogen activator, urokinase), which itself has 10 high-confidence associations to other genes, none of which is related to type A muscular dystrophy-dystroglycanopathy. Therefore, although mutations in *TMEM5* cause the same disease as mutations in the other genes of this family (*POMGNT1*, *POMGNT2*, *ISPD*, *FKTN*, *POMT1*, *POMT2*, *FKRP*, *LARGE*), there is little functional similarity reflecting this in STRING. Thus, although PPA analysis offers an effective way of prioritizing disease genes in many cases, there are disease genes that do not show a high random walk score.

In cases where the causative gene does not interact with previous members of the disease-gene family, or for diseases where there are no previously known genes, other approaches will have to be considered. We recently described an approach, Exomiser, that uses phenotype comparisons with model organism data to inform on candidacy (Robinson *et al.*, 2014). eXtasy is another recently published solution that uses phenotype comparisons along with consideration of many other data types (Sifrim *et al.*, 2013). To contrast and compare these different approaches we applied them to the same set of recently solved cases and report the performance in Table 1. Note that eXtasy does not perform any variant filtering, and so, to allow a fair comparison we used VCF files that had already been filtered in the same way as for the ExomeWalker benchmarking. Three of the diseases currently have no phenotype annotations available and are therefore not runnable through eXtasy or particularly amenable to Exomiser prioritization. eXtasy can only inform on non-synonymous variants and four of the cases involve a small deletion, which again was not assessable. Finally, for two of the cases, eXtasy removed the causative variant during analysis, so no final ranking was possible. For 2 of the 10 remaining cases, Exomiser and eXtasy performed better than ExomeWalker, with ExomeWalker outperforming them in the other cases. *KLHL3* is a good example where there is minimal evidence for interactions with previously implicated genes but where use of phenotype data allowed identification of the causative variant as the top or second best hit using eXtasy or Exomiser, respectively. In contrast for the three cases where ExomeWalker identified the causative gene as the top hit, both eXtasy and Exomiser were unable to achieve this efficient prioritization.

## 5 CONCLUSION

We have implemented our method in a freely available Web server called ExomeWalker. Users can upload a VCF file and choose one of 243 phenotypic series or enter their own disease-gene family in the form of a list of Entrez Gene identifiers. These genes may already be known to be associated with the disease or be members of a pathway suspected of being disrupted in the disease or just candidates from in-house knowledge. If the VCF file contains multiple samples, ExomeWalker will assume that all samples are from the same family and will ask the user to upload a pedigree (PED) file. It will then perform pedigree filtering on the genes and variants represented in the VCF file using the Jannovar library (Jäger *et al.*, 2014). It will subsequently rank the candidate genes and return a list of candidates together with information about the genes. Importantly, it will show all first- and second-degree interactions with the seed genes, allowing users to quickly eyeball candidate lists to determine if there are genes with multiple functional associations with the seed genes that would reward closer inspection. Exome sequencing remains a difficult endeavor, and large-scale exome-sequencing studies for the identification of Mendelian disease causing genes have reported success rates around 20–35% (de Ligt *et al.*, 2012; Yang *et al.*, 2013). Therefore, it is not realistically to be expected that a prioritization method will place the correct gene in the first place, or first few places, in all cases. An advantage of the methodology presented here is that ExomeWalker quickly shows whether there are candidate genes with both predicted pathogenic variants and multiple functional associations with other genes in the same disease-gene family. If this is not the case, users may wish to explore phenotype-based (Robinson *et al.*, 2014) or genomic data fusion (Sifrim *et al.*, 2013) prioritization of exome data, or if possible sequence additional family samples to enable linkage filtering (Rödelsperger *et al.*, 2011; Smith *et al.*, 2011), or sequence additional unrelated individuals for intersection-based (Robinson *et al.*, 2011) approaches.

The ExomeWalker server is freely available at http://compbio.charite.de/ExomeWalker/.

*Funding*: This work was supported by grants of the Bundesministerium für Bildung und Forschung (BMBF project number 0313911), by the European Community’s Seventh Framework Programme (Grant Agreement 602300; SYBIL) and by core infrastructure funding from the Wellcome Trust. Additional support was provided by the Director, Office of Science, Office of Basic Energy Sciences, of the US Department of Energy under contract no. DE-AC02-05CH11231.

*Conflict of interest*: none declared.
